# Integrin alpha 6 homozygous splice-site mutation causes a new form of junctional epidermolysis bullosa in Charolais cattle

**DOI:** 10.1186/s12711-023-00814-1

**Published:** 2023-06-12

**Authors:** Mekki Boussaha, Arnaud Boulling, Valérie Wolgust, Lorraine Bourgeois-Brunel, Pauline Michot, Cécile Grohs, Nicolas Gaiani, Pierre-Yves Grivaud, Hélène Leclerc, Coralie Danchin-Burge, Marthe Vilotte, Julie Rivière, Didier Boichard, Jean-Marie Gourreau, Aurélien Capitan

**Affiliations:** 1 Université Paris-Saclay, INRAE, AgroParisTech, GABI, 78350 Jouy-en-Josas, France; 2 Unité de Pathologie du Bétail, Ecole Nationale Vétérinaire d’Alfort, 94700 Maisons-Alfort, France; 3 Eliance, 75012 Paris, France; 4 Cabinet Vétérinaire des Monts du Charolais, 71220 Saint Bonnet de Joux, France; 5 Institut de l’Elevage, 75012 Paris, France; 6 Université Paris-Saclay, INRAE, AgroParisTech, MICALIS, 78350 Jouy-en-Josas, France; 7 Present Address: Herd Book Charolais, 58470 Magny-Cours, France

## Abstract

**Background:**

Inherited epidermolysis bullosa (EB) is a group of painful and life-threatening genetic disorders that are characterized by mechanically induced blistering of the skin and mucous membranes. Congenital skin fragility resembling EB was recently reported in three Charolais calves born in two distinct herds from unaffected parents. Phenotypic and genetic analyses were carried out to describe this condition and its molecular etiology.

**Results:**

Genealogical, pathological and histological investigations confirmed the diagnosis of recessive EB. However, the affected calves showed milder clinical signs compared to another form of EB, which was previously reported in the same breed and is caused by a homozygous deletion of the *ITGB4* gene. Homozygosity mapping followed by analysis of the whole-genome sequences of two cases and 5031 control individuals enabled us to prioritize a splice donor site of *ITGA6* (c.2160 + 1G > T; Chr2 g.24112740C > A) as the most compelling candidate variant. This substitution showed a perfect genotype–phenotype correlation in the two affected pedigrees and was found to segregate only in Charolais, and at a very low frequency (f = 1.6 × 10^−4^) after genotyping 186,154 animals from 15 breeds. Finally, RT-PCR analyses revealed increased retention of introns 14 and 15 of the *ITGA6* gene in a heterozygous mutant cow compared with a matched control. The mutant mRNA is predicted to cause a frameshift (ITGA6 p.I657Mfs1) that affects the assembly of the integrin α6β4 dimer and its correct anchoring to the cell membrane. This dimer is a key component of the hemidesmosome anchoring complex, which ensures the attachment of basal epithelial cells to the basal membrane. Based on these elements, we arrived at a diagnosis of junctional EB.

**Conclusions:**

We report a rare example of partial phenocopies observed in the same breed and due to mutations that affect two members of the same protein dimer, and provide the first evidence of an *ITGA6* mutation that causes EB in livestock species.

**Supplementary Information:**

The online version contains supplementary material available at 10.1186/s12711-023-00814-1.

## Background

Junctional epidermolysis bullosa (JEB) is a rare autosomal recessive genodermatosis that is characterized by mechanically induced blistering of the skin and mucous membranes. Tissue cleavage occurs in the lamina lucida layer of the epidermal basement membrane secondary to mutations in genes that encode components of the hemidesmosome anchoring complex (COL17A1, ITGA6, ITGB4, LAMA3, LAMB3, and LAMC2) [1]. This condition has been reported to adversely affect the health and survival of affected individuals in different species, including cattle and humans [2–9]. Due to the lack of curative treatments, most human JEB patients die within the first year of life from malnutrition and various medical complications [10], while affected livestock are usually euthanized at birth.

In 2015, we described a large deletion in the *integrin subunit beta 4* (*ITGB4*) gene that causes severe JEB among the inbred descendants of a Charolais bull [3]. Since then, three purebred Charolais calves with clinical signs reminiscent of a milder form of JEB, and which turned out to be homozygous wildtype for the latter variant (see after), were referred to the French National Observatory for Bovine Abnormalities (ONAB; https://www.onab.fr/). The purpose of this study was to characterize this new genetic defect in Charolais cattle.

## Methods

### Animals

Three male calves affected by congenital skin fragility were observed within a period of 5 years on two commercial farms that are more than 300 km distant from each other and with no obvious link (i.e. no recent history of trade of reproducers between them or with a common herd). Only one of the breeders kept genealogical records, which were extracted from the French national pedigree database. The three affected calves were euthanized by intravenous injection of T61 (MSD France) and subjected to a complete post-mortem examination. Oral mucosa and skin from various regions of the body were sampled for histological examinations. In addition, the tip of one pinna was collected from each animal for DNA extraction. At the time of the study, biological samples for DNA extraction were also available for the three dams of the cases, one of their sire, and one of their unaffected half-brother. In addition, ear biopsies from one of the dams that were heterozygous for the *ITGA6* splice-site variant and one wild type control were collected at slaughter and conserved at – 80 ℃ for subsequent RNA extraction (see below).

### Histology

Skin samples were fixed in 10% neutral buffered formalin for 24 h at + 4 ℃ and then dehydrated through a graded ethanol series, cleared with xylene and embedded in paraffin wax. Microtome section (5 µm, Leica RM2245) were mounted on adhesive slides (Klinipath- KP-PRINTER ADHESIVES), deparaffinized, and stained with haematoxylin, eosin and saffron (HES) or with periodic acid-Schiff (PAS). Slides were scanned with the Pannoramic Scan 150 (3D Histech) and analyzed with the CaseCenter 2.9 viewer (3D Histech).

### DNA extraction

DNA was extracted from blood or ear samples using the DNeasy Blood and Tissue Kit (Qiagen). DNA quality was controlled by electrophoresis and quantified using a Nanodrop spectrophotometer (Thermo Scientific).

### Mapping of the new JEB locus

The three affected calves, five of their close relatives (see section “Animals”), as well as 10,914 controls from the Charolais breed were genotyped with different single nucleotide polymorphism (SNP) arrays over time (Illumina Bovine SNP50, EuroG10K and EuroGMD). Genotypes were phased and imputed to the Bovine SNP50 using the FImpute3 software [11] within the framework of the French genomic evaluation, as described in [12]. Assuming a monogenic recessive inheritance, we searched for identical-by-descent (IBD) haplotypes of at least 20 SNPs (~ 1 Mb) that were in the homozygous state in the three cases and found only in the heterozygous state and at a low frequency in the control group.

### Analysis of whole-genome sequences

The genomes of affected calves #2 and #3 were sequenced at a coverage of 17 and 23 × with the Illumina HiSeq technology in 101 and 150 paired-end mode, respectively, after library preparation using the NEXTflex PCR-Free DNA Sequencing Kit (Bioscientific). Reads were aligned on the ARS-UCD1.2 bovine genome assembly and processed in accordance with the guidelines of the 1000 Bull Genomes Project [13] for variant detection. Only SNPs and small Indels located within the mapping interval (positions 18,367,448 to 24,692,900 bp on chromosome 2) and for which both cases were homozygous for the alternative allele were considered. Additional genomes from run 9 of the 1000 Bull Genomes Project were used for filtering purpose. They consisted of 153 control Charolais cattle, which were expected to be either heterozygous or homozygous wild-type for the new JEB variant, and 4878 individuals from various genetic backgrounds free of Charolais ancestry and which were assumed to be homozygous wild-type. The remaining variants were annotated using Variant Effect Predictor (Ensembl release 106; https://www.ensembl.org/info/docs/tools/vep/index.html). We focused on the following categories of annotations: substitutions with a SIFT score ≤ 0.05, premature start, start loss, stop loss, stop gain, frameshift, inframe insertion or deletion, and splice donor or acceptor site.

In addition, we detected structural variants (SV) within the mapping interval using Pindel [14], Delly [15] and Lumpy software [16] and applied the same filters after comparison with analogous catalogs of SV of 200 control genomes reported in [17, 18].

### Genotyping of the *ITGB4* deletion and *ITGA6* splice site variant

The three JEB-affected calves, their dams, one of their sires and one unaffected male halfsib were genotyped for the *ITGB4* deletion by PCR and agarose gel electrophoresis (as described in [3]) and for the *ITGA6* splice site variant (g.24112740C > A on chromosome 2) by PCR and Sanger sequencing. A 518-bp segment encompassing this substitution was amplified using a Mastercycler Pro thermocycler (Eppendorf) with primer pair ACTGGCTGTGTTTTCACGA/CCAAAGAATCCCACCAAAGA and the GoTaq Flexi DNA Polymerase kit (Promega), following the manufacturer’s instructions. Amplicons were purified and sequenced on both strands by Eurofins MWG (Hilden, Germany). Finally, variant calling was performed with the novoSNP software [19].

In addition, to genotype the *ITGA6* splice variant on a large scale, we added a probe to the Illumina EuroGMD SNP array using the following design: TCACTTACCAGGCAATGTTGTTTTTTTTTTCCCCTCCGAGCTGGTCTCTTAAAATACTNN[A/C]AGGGAAAGCCCTCAGTTCTCTATATGCAGAGTAAGTCAGAGTGTCTGGAAAAGTGGCAAT The EuroGMD SNP array is routinely used for genomic evaluation in France and genotypes of 186,154 animals from 15 breeds were available for this variant.

### RT-PCR analysis of *ITGA6* mRNA

RNA was extracted from ear punch biopsies (4-mm diameter) using the RNeasy Mini Kit (Qiagen). Thirty mg of tissue were briefly homogenized in RLT buffer with a T 25 digital Ultra-Turrax machine (IKA laboratories) and then total RNA was obtained by following the manufacturer’s instructions. RNA concentration and purity were evaluated using a Nanodrop One spectrophotometer (Thermo Fisher Scientific). A commercial sample of total RNA from bovine muscle (Gentaur) was used as a positive control. Then, 40 ng of RNA were reverse-transcribed using the SuperScript^®^ III First-Strand Synthesis System for RT-PCR (reverse transcription-polymerase chain reaction; Invitrogen) using oligo (dT). One µL of cDNA was PCR-amplified using a Mastercycler Pro thermocycler (Eppendorf), with the GoTaq Flexi DNA Polymerase kit (Promega) and forward and reverse primers located on exon 14 and 16, respectively (5′-GTGCACTTCTTAAAAGAGGGATG-3′, 5′-TTTTAAAAGGATTCCCAAGCTC-3′). PCR amplicons were visualized on 2% low-melting agarose gel electrophoresis stained with ethidium-bromide in 1 × TBE buffer. Finally, bright DNA bands were gel-purified with the QIAquick Gel Extraction Kit (Qiagen) and bidirectionally sequenced by Eurofins MWG (Hilden, Germany) using Sanger’s method.

### Prediction of the consequences of mutation on the encoded protein

A mutant mRNA sequence was created by inserting introns 14 and 15 into the wild type *ITGA6* cDNA using information from Ensembl release 106 (Entry ENSBTAT00000085126.1; www.ensembl.org). This mutant cDNA was translated using ExPASy translate tools (http://us.expasy.org/translatetool/). Predictions of the integrin alpha2 domain and topologies were obtained with UniProt (https://www.uniprot.org/) and DeepTMHMM (https://dtu.biolib.com/DeepTMHMM), respectively.

## Results and discussion

### Pathological and genealogical investigations

**Fig. 1:**
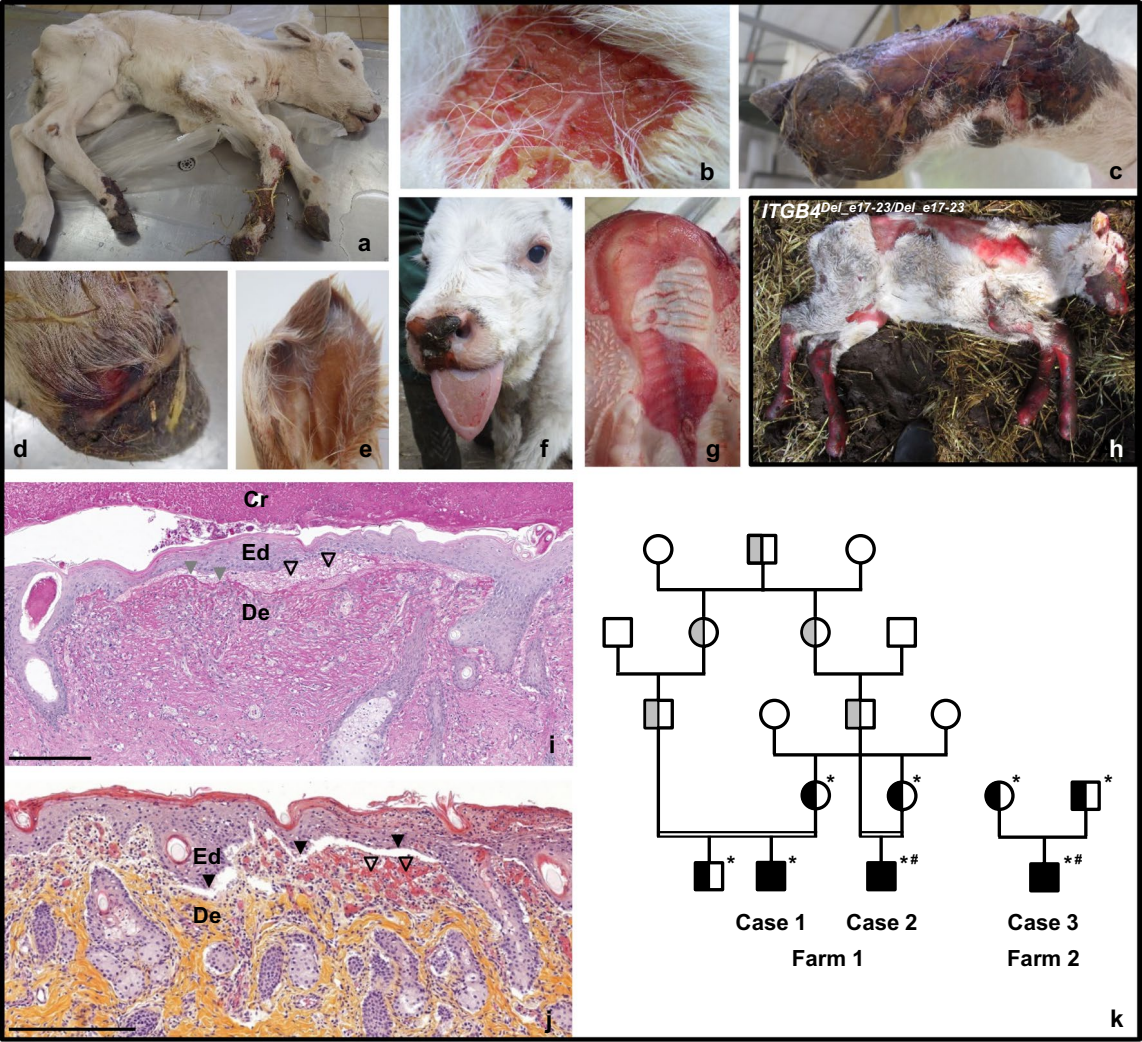
Pathological features and pedigree of three Charolais calves affected by a new form of congenital skin fragility. General view of case #2 **(a)** and details of a lesion at the basis of the neck (**b**), of the extremity of the right posterior limb **(c**, **d**), and of the left ear (**e**). Pictures of case #3 showing lesions on the muzzle, tongue **(f**) and hard palate (**g**). **h)** *ITGB4*^Del_e17−23/Del_e17−23^ calf affected by a severe form of JEB previously reported in the same breed. Histological sections of the skin of the knee at the level of a lesion (**i**) and of the external skin of the cheek in a grossly normal area (**j)** from case #2 stained with PAS and HES, respectively. *Ed* epidermis, *Cr *crust, *De *dermis. Black arrowheads point to areas of sub-epithelial splitting and blistering, empty arrowheads indicate inflammatory infiltrates, and grey arrowheads highlight the basement membrane. Bars = 200 μm. **k)** Pedigree of the affected calves and their relatives. Filled and semi-filled symbols represent affected and carrier animals, respectively, based on direct genetic testing (black) or inferred from pedigree information (grey). *Animals with DNA samples available. #: Individuals selected for whole genome sequencing

Three male Charolais calves that were affected by congenital skin fragility were born after full-term gestation on two independent farms (Fig. 1a). Because of poor prognosis they were euthanized in their first week of life and necropsied. Pathological investigations revealed skin erosion and ulceration in areas of skin folds (Fig. 1b) and friction (Fig. 1c), early signs of dysungulation (Fig. 1c, d), and slight malformation of the tip of the pinna (Fig. 1e). Affected animals also showed oral mucosal blistering (Fig. 1f, g), which was consistent with breeders’ reports of pain on suckling and feeding difficulties. No macroscopic lesions were observed in the rest of the digestive system or other internal organs. Overall the pathological features observed in these three calves were milder than those of a recessive form of JEB that was previously reported in the same breed (Fig. 1a vs Fig. 1h; [3, 5]). Indeed, calves that were homozygous for a deletion of exons 17 to 23 in the *ITGB4* gene (hereafter referred to as “ITGB4Del_e17-23/Del_e17-23”) showed multifocal skin ulcers, atrophied external ears and complete lack of hoof horn from birth. Histopathological examinations revealed sub-epithelial splitting under the basal keratinocytes of the epidermis and above the periodic acid Schiff (PAS)-positive basement membrane (Fig. 1i), as well as dermal inflammatory infiltrates in some of the skin sections (Fig. 1i, j). Two of the affected calves were born on the same farm from unaffected and closely-related parents, which supports autosomal recessive inheritance. Unfortunately, no genealogical record was available for the third case that was reported in a distinct herd (Fig. 1k). Interestingly, genotyping by PCR and Sanger sequencing of the three affected calves for the *ITGB4* causal variant revealed that they were all homozygous wildtype. This led us to consider their condition as a new recessive form of EB in Charolais.

### Mapping and identification of a candidate variant in the *ITGA6* gene

To gain insights into the molecular basis of this new genetic defect, we conducted a series of genetic analyses. First, we applied a homozygosity mapping approach using phased and imputed Illumina BovineSNP50 genotypes. We identified a 150-marker haplotype on chromosome 2 (from position 18,380,242 bp to 24,654,383 bp on the bovine reference genome assembly ARS-UCD1.2 [20]) that was: (i) shared by the parental phases of the three cases and (ii) never observed in the homozygous state in 10,914 controls. The most proximal markers located outside of this IBD segment defined a 6.3-Mb mapping interval (Chr2:18,367,448–24,692,900 bp).

**Fig. 2:**
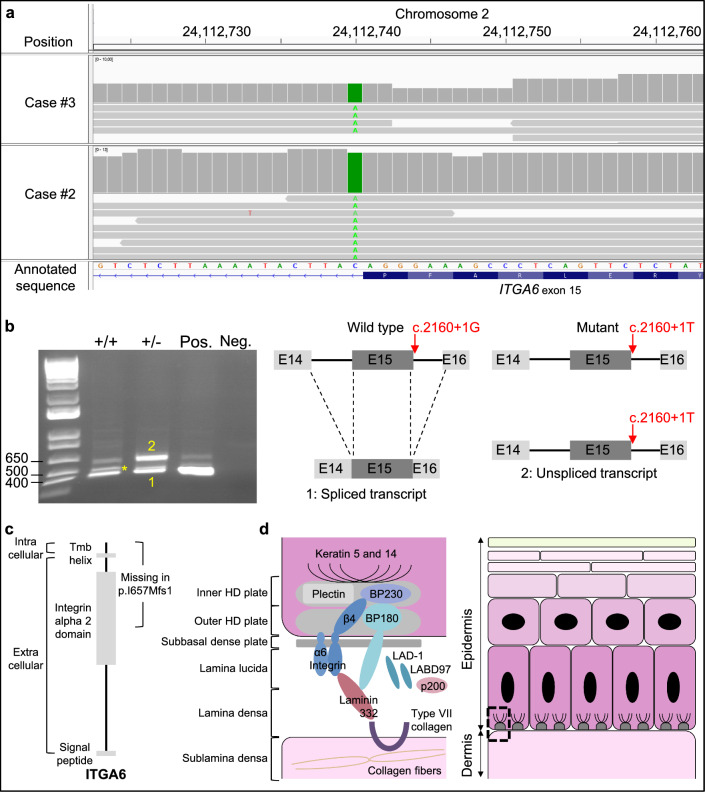
Identification of a splice site mutation in the *ITGA6* gene and characterisation of its effects. **a**) IGV screenshot showing read coverage (up) and sequences (down) for cases #2 and #3 around variant Chr2 g.24112740C > A (*ITGA6* c.2160 + 1G > T). **b)** Analysis of the consequences of this variant on the splicing of *ITGA6*. Left) Agarose gel electrophoresis after RT-PCR on negative control (Neg., water), positive control (Pos., commercial bovine muscle RNA), and ear biopsies RNA from one homozygous wild type (+/+) and one heterozygous carrier (+/-) of the mutation. *note the presence of a third band corresponding to partially spliced transcripts following the excision of either intron 14 or intron 15. Right) Details for the amplified segments after verification using Sanger sequencing. Boxes E14, E15, E16 correspond to exons 14 to 16. **c)** scheme of the wild type (Wt) and mutant ITGA6 proteins with domain information. Tmb Helix) transmembrane Helix. **d)** Structure of the hemidesmosomes (HD) and the dermo-epidermal zone (adapted from [27–29]). Note that BP180 is also known as COL17A1 and that laminin 332 is composed of LAMA3, LAMB3, and LAMC2

Then, we sequenced the complete genomes of one case from each farm. After filtering homozygous variants shared by these two EB-affected calves with information from 5031 control genomes from various breeds, we reduced the list of candidates to only eight SNPs (see Additional file 1: Table S1). Among these SNPs, only one was predicted to be deleterious, i.e. a substitution affecting the first nucleotide of the splice donor site of exon 15 of the gene encoding the integrin alpha 6 subunit (*ITGA6* c.2160 + 1G > T; Chr2 g.24112740C > A; Fig. 2a). For verification, first we genotyped this variant by PCR and Sanger sequencing in the two affected pedigrees and found a perfect genotype–phenotype correlation. The three cases were homozygous for allele c.2160 + 1 T, while the four parents investigated and an unaffected halfsib were heterozygous. Then, we performed large-scale genotyping among 186,154 animals from 15 breeds using the Illumina EuroGMD SNP array (Table 1). We observed only two heterozygous carriers of the c.2160 + 1 T allele in Charolais cattle indicating that this variant is breed-specific and very rare (f = 1.6 × 10^–4^). Further analysis of the pedigree of the 6254 Charolais animals genotyped showed that the derived allele segregated at a frequency of 0.4% in the natural mating population (two unrelated carriers out of 487 individuals descended from 280 natural service sires and 329 natural service maternal grandsires). In contrast, the variant was absent in 5767 animals (descended from 505 sires and 764 maternal grandsires) that had at least one artificial insemination bull among their first and second-generation ancestors.

**Table 1:** Genotypes of 186,154 animals from 15 bovine breeds for the *ITGA6* splice donor variant (Chr2 g.24112740C > A; c.2160 + 1G > T)

Breed	CC	AC	AA
Abondance	2329	–	–
Aubrac	12	–	–
Blonde d’Aquitaine	2855	–	–
Brune	2132	–	–
Charolais	6252	2	–
Holstein	100,647	–	–
Jersey	1047	–	–
Limousine	690	–	–
Montbéliarde	52,132	–	–
Normande	13,257	–	–
Parthenaise	333	–	–
Salers	422	–	–
Simmental	2441	–	–
Tarentaise	1252	–	–
Vosgienne	351	–	–

### Consequences of *ITGA6* c.2160 + 1G > T on the mRNA transcript sequence

To investigate the effects of the c.2160 + 1G > T splice-site mutation on the ITGA6 protein, we performed immunohistochemical analyses on paraffin-embeded skin sections from homozygous mutant and control individuals. In the absence of commercial antibodies directed against the bovine protein, we used one mouse anti-human monoclonal antibody that targets the N-terminal region (reference sc-374057, Santa Cruz), and one rabbit anti-human polyclonal antibody that targets the C-terminal region of the protein (LS-C168548, LifeSpan BioSciences). Unfortunately, we were unable to detect a specific signal at the expected tissular localization in wildtype samples (results not shown). We assume that these two antibodies do not work with the bovine orthologous protein due to small differences in amino-acid sequence with the human recognized epitopes.

We also carried out RT-PCR analyses using primers that are located within exons 14 and 16 in order to investigate the impact of variant c.2160 + 1G > T on the splicing of *ITGA6* mRNA in the ear skin from one wildtype (+/+) and one heterozygous (+/-) cow (Fig. 2b). Agarose gel electrophoresis and Sanger sequencing revealed the existence of two main bands for both animals, corresponding to a correctly spliced transcript (i.e. containing exons 14, 15 and 16; band 1 on Fig. 2) and to a unspliced transcript still carrying introns 14 and 15 (band 2 on Fig. 2). Intermediate weak products (asterisk on Fig. 2) that carried probably only one intron were also observed but not sequenced. Interestingly, while the correctly spliced transcript represented a majority of the amplicons in the control animal, bands 1 and 2 were of equal intensity for the heterozygous animal. This observation suggests that the c.2160 + 1G > T substitution causes a dramatic decrease in the excision of introns 14 and 15, and it is reasonable to assume that the level of normal *ITGA6* transcript is low or even null in homozygous carriers of the mutant allele.

The retention of both introns 14 and 15 in *ITGA6* transcripts is predicted to lead to a frameshift generating a premature termination codon (ITGA6 p.I657Mfs1, Fig. 2c). The resulting product would lack 40% of the amino acid sequence including half of its integrin alpha 2 domain and the entirety of the transmembrane domain. This mutation is predicted to affect both the assembly of the integrin α6β4 dimer and its correct anchoring to the cell membrane. This dimer is a key component of the hemidesmosome anchoring complex, which ensures the attachment of basal epithelial cells to the basal membrane (Fig. 2d). As mentioned in the Background section, mutations in ITGA6 and five other proteins of this complex (COL17A1, ITGB4, LAMA3, LAMB3, and LAMC2) have been previously reported to cause JEB in humans and mouse [1, 21–26]. However to date, based on a review of the literature in PubMed (https://pubmed.ncbi.nlm.nih.gov/) and Online Mendelian inheritance in animals (https://omia.org/home/), no mutation in the *ITGA6* gene has been reported in non-model animal species. Based on all these elements, we arrived at a diagnosis of JEB.

Our study provides interesting information on the pathological features that are associated with a recessive mutation of the *ITGA6* gene in cattle and it expands the list of spontaneous models available in livestock species for syndromes that are also observed in humans. In addition, after our previous report of a large deletion in the *ITGB4* gene in Charolais [3, 4], the identification of a splice site variation in *ITGA6* that is responsible for a milder form of JEB, represents a rare example of partial phenocopies observed in the same breed and due to mutations affecting two members of the same protein dimer. The difference in severity between these two forms of JEB might be due to the existence of low levels of normal *ITGA6* transcript in homozygous mutants, possibly after natural RNA edition. Unfortunately, we could not verify this hypothesis because immunohistochemical analyses did not work, and tissues collected from affected animals at the time of their death (i.e., before the discovery of the *ITGA6* c.2160 + 1G > T variant) were not available for a posteriori RNA extraction due to their initial processing (fixed in formalin or used for DNA extraction).

## Conclusions

In conclusion, we report a rare example of genetic heterogeneity for a sporadic recessive genetic defect within a single cattle breed and provide the first evidence of a mutation in the *ITGA6* gene that causes JEB in livestock species. The development of a diagnostic test and the low frequency of the mutant allele in the Charolais breed will enable its efficient counterselection.

## Supplementary Information

**Additional file 1: Table S1. **List of candidate variants for a new recessive form of JEB in Charolais cattle.

## Data Availability

The whole-genome sequences of two JEB-affected calves have been deposited in the European Nucleotide Archive (ENA) at EMBL-EBI under the study accession number PRJEB47654.
